# The social patterning of risk factors for noncommunicable diseases in five countries: evidence from the modeling the epidemiologic transition study (METS)

**DOI:** 10.1186/s12889-016-3589-5

**Published:** 2016-09-09

**Authors:** Silvia Stringhini, Terrence E. Forrester, Jacob Plange-Rhule, Estelle V. Lambert, Bharathi Viswanathan, Walter Riesen, Wolfgang Korte, Naomi Levitt, Liping Tong, Lara R. Dugas, David Shoham, Ramon A. Durazo-Arvizu, Amy Luke, Pascal Bovet

**Affiliations:** 1 University Institute of Social and Preventive Medicine (IUMSP), Lausanne University Hospital, Biopôle 2, Route de la Corniche 10, 1010 Lausanne, Switzerland; 2 Tropical Medicine Research Institute, University of the West Indies, Mona, Kingston Jamaica; 3 Kwame Nkrumah University of Science and Technology, Kumasi, Ghana; 4 Research Unit for Exercise Science and Sports Medicine, University of Cape Town, Cape Town, South Africa; 5 Ministry of Health, Victoria, Republic of Seychelles; 6 Center for Laboratory Medicine, Canton Hospital, St. Gallen, Switzerland; 7 Chronic Disease Initiative in Africa, Department of Medicine, University of CapeTown, Cape Town, South Africa; 8 Stritch School of Medicine, Loyola University Chicago, Maywood, IL USA

**Keywords:** Socioeconomic status, Noncommunicable diseases, Low and middle income countries, Smoking, Physical activity, Obesity, Hypertension, Risk factors

## Abstract

**Background:**

Associations between socioeconomic status (SES) and risk factors for noncommunicable diseases (NCD-RFs) may differ in populations at different stages of the epidemiological transition. We assessed the social patterning of NCD-RFs in a study including populations with different levels of socioeconomic development.

**Methods:**

Data on SES, smoking, physical activity, body mass index, blood pressure, cholesterol and glucose were available from the Modeling the Epidemiologic Transition Study (METS), with about 500 participants aged 25–45 in each of five sites (Ghana, South Africa, Jamaica, Seychelles, United States).

**Results:**

The prevalence of NCD-RFs differed between these populations from five countries (e.g., lower prevalence of smoking, obesity and hypertension in rural Ghana) and by sex (e.g., higher prevalence of smoking and physical activity in men and of obesity in women in most populations). Smoking and physical activity were associated with low SES in most populations. The associations of SES with obesity, hypertension, cholesterol and elevated blood glucose differed by population, sex, and SES indicator. For example, the prevalence of elevated blood glucose tended to be associated with low education, but not with wealth, in Seychelles and USA. The association of SES with obesity and cholesterol was direct in some populations but inverse in others.

**Conclusions:**

In conclusion, the distribution of NCD-RFs was socially patterned in these populations at different stages of the epidemiological transition, but associations between SES and NCD-RFs differed substantially according to risk factor, population, sex, and SES indicator. These findings emphasize the need to assess and integrate the social patterning of NCD-RFs in NCD prevention and control programs in LMICs.

## Background

Surveillance of risk factors of non communicable diseases (NCD-RFs) is an essential component of programs to address non-communicable diseases (NCDs) in all countries [1]. It is well recognized that socioeconomic status (SES) influences the risk and vulnerability of individuals to NCD-RFs and NCDs, which underlies the need to examine the prevalence of NCD-RFs along SES categories. While the social patterning of NCD-RFs is well documented in high income countries, fewer studies are available in low and middle income countries (LMICs), and findings are generally inconsistent.

One potential explanation for mixed results on the social patterning of NCD-RFs in LMICs is the fact that the direction of these associations may change along a country’s socioeconomic development [2–6]. A recent study using data from the World Health Survey showed that the prevalence of smoking was higher and consumption of fruit and vegetables lower among lower SES groups in high income as well as in LMICs [7]. On the other hand, physical inactivity was less prevalent in populations of low SES, especially in low-income countries, and results were mixed for alcohol consumption [7]. A recent review suggested that, in LMICs, high SES is associated with overall healthier dietary patterns but also with higher calorie, cholesterol, and saturated fat intakes [8]. Blood concentration of total cholesterol and the prevalence of obesity, hypertension and diabetes are generally higher among individuals with high SES in low-income countries [3, 4, 9, 10], while in middle income countries they are generally higher among low SES individuals [2, 11, 12].

Although evidence is accumulating for a “social transition” of NCD-RFs from the higher to the lower SES groups along a country’s socioeconomic development [2–6], this notion has been vividly debated recently [13, 14]. The importance of the socio-economic determinants of NCDs has been frequently highlighted in recent years [15, 16]. However, few studies examined the social patterning of NCD-RFs in LMICs and direct comparison of results is often limited by the different methods used in different studies. In this study, we examine the association of education and wealth with several NCD-RFs (smoking, physical activity, alcohol abuse, obesity, hypertension, high cholesterol and high blood glucose) in young adults (25–45 years) in five populations of African descent at different stages of the epidemiological transition, drawn from Ghana, Jamaica, South Africa, Seychelles and the United States.

## Methods

### Study population

A detailed description of the METS study has been previously published [13]. In brief; 500 participants, approximately 50 % female, were enrolled in each of five study sites: rural Ghana, urban Jamaica, urban South Africa, the Seychelles and Maywood (suburb of Chicago, IL, USA). These sites represent a broad range of socio-economic development, as defined by the United Nations Human Development Index (HDI), i.e., low-middle for Ghana, middle for South Africa, high for Jamaica and the Seychelles, and very high for the US. For recruitment; a simple random sample was generated in Ghana from the population census for the rural town of Nkwantakese; a sex- and age-stratified random sample was drawn from previously enumerated areas of Khayelitsha in South Africa (a large township adjacent to the city of Cape Town); districts were randomly sampled in Kingston, Jamaica, beginning from a fixed point in each district with door-to-door recruitment; a sex- and age-stratified random sample was used in Seychelles from the national census; and all city blocks in the community were randomized and door-to-door recruitment was conducted in Maywood, USA (suburbs of Chicago). Individuals were excluded if they were HIV-positive, were pregnant or lactating, or were unable to participate in normal physical activities. METS was approved by the institutional ethics committees in the five sites involved. Written informed consent was obtained from all participants.

### Measures

All measurements were performed early in the morning at outpatient clinics or testing sites, located in the communities. *Smoking* was assessed by an administered structured questionnaire. Current smokers refer to participants reporting smoking at least one cigarette per day. *Physical activity* was measured using an accelerometer (Actical, Phillips Respironics, Bend, OR, USA), with participants wearing the accelerometer at all times during 8 days (apart while bathing, showering, or swimming). Data were obtained based on six complete days of activity. Weight was measured to the nearest 0.1 kg using the same standard calibrated weighing scale at all 5 sites (Seca 770, Hamburg, Germany). Height was measured to the nearest 0.1 cm using a stadiometer (Invicta Stadiometer, Invicta, London, UK) with the participant’s head held in the Frankfort plane. We used weight and height to calculate body mass index (BMI,kg/m^2^). *Obesity* was defined as BMI ≥ 30 kg/m^2^.

Systolic and diastolic blood pressures were measured using an automatic digital blood pressure monitor in all sites (HEM-747Ic, Omron Healthcare, Bannockburn, IL, USA). Six measurements were made in two sets of three; the mean of the last 2 measurements for each set is considered. *Hypertension* was defined as systolic/diastolic BP ≥ 140/90 mmHg. Participants were asked to fast from the evening prior to the baseline clinic examination. Fasting blood samples were drawn for analysis of glucose and lipids. Serum was obtained within two hours of collection and stored at −80 °C at each study site. Analyses of lipids were conducted at the Center for Laboratory Medicine, Canton Hospital, St. Gallen, Switzerland. *High cholesterol* was defined as total cholesterol ≥5.2 mmol/l. Fasting plasma glucose was measured using the glucose oxidase method at each site at the time of collection. *Elevated blood glucose* was defined as plasma glucose ≥5.6 mmol/l in all sites. In the Ghanaian sample we did not examine elevated blood glucose as there was no certainty that participants were in the fasting state at blood collection.

### Socio-economic indicators

Two indicators of SES were used: education and a proxy measure of wealth (referred as “wealth” in this paper). Education was assessed with the question: “Do you have any of the following degrees?” and was categorized as high (post secondary and tertiary education, corresponding to the International Standard Classification of Education (ISCED) levels 4–8), middle (secondary or vocational education, ISCED levels 2–3) and low (primary education, ISCED levels 0–1) [17]. Wealth was calculated from several questions assessing the household’s ownership of a number of assets (iron, fridge, cable, etc.), categorized as 0/1. These items were summed and an assets ownership score was created; the score was further categorized according to site-specific tertiles.

### Statistical analysis

Statistical analysis was conducted using Stata v.13.1 (Stata corp, College Station, TX, USA). With the few exceptions mentioned below, all analyses were stratified by site. Because there was no evidence of gender differences in the effect of education or wealth on most NCD-RFs (p for interaction >0.05) and given the small sample size, main analyses were sex-adjusted. Sex-stratified analyses are presented as supplements. We used least squares linear regression to calculate age-and sex-adjusted prevalence rates of NCD-RFs for each educational/wealth group in the five sites. Relative inequalities in NCD-RFs were assessed using age-and sex-adjusted logistic regressions, separately for education and wealth. In order to test whether the associations between SES and NCD-RFs differed by site, an interaction term between education or wealth and site was fitted in the different regression models described above including all sites. We further tested whether education modified the association between wealth and NCD-RFs by adding an interaction term between education and wealth in the logistic regression analyses.

## Results

Of the 2506 participants originally included in the METS study, 326 were excluded from the present analysis because of missing values for SES indicators (*N* = 9) or NCD-RFs (*N* = 326), categories not mutually exclusive. Excluded participants were more likely to be men (*p* < 0.001), had lower education but higher wealth (*p* < 0.001) and were more frequently from Jamaica or the USA (*p* < 0.001). There were no age differences between excluded and included participants.

Table 1 shows the basic characteristics of the 2180 participants (55 % women) included in the present study. 26.2 % were current smokers overall, the prevalence of smoking being highest in the USA sample (46.9 %) and lowest in the Ghanaian sample (2.5 %), with large gender differences. The same pattern was observed for obesity (31.7 % overall, 53.1 % in the USA sample and 10.4 % in the Ghanaian sample). About 40 % of participants were physically active. Hypertension was very low in the Ghanaian and Jamaican samples (prevalence <10 %) and high in the USA sample (21.4 %). The prevalence of high cholesterol and elevated blood glucose were lowest in the South African sample (10.7 %/9.1 %, respectively) and highest in the USA sample (25.0 %/41.1 %, respectively).

**Table 1:** Characteristics of the participants included in the study by site

	Total	Ghana	South Africa	Jamaica	Seychelles	USA	*P* ^*a*^
N	2180	441	485	368	438	448	
GNP^b^ ($)		1′260	6′100	4′550	10′270	48′960	
HDI^cd^		0.621	0.540	0.727	0.799	0.934	
World bank income class^d^		Lower middle income	Upper middle income	Upper middle income	High income	High income	
Women, N (%)	1209 (55.5)	265 (60.1)	257 (53.0)	227 (61.8)	229 (52.3)	231 (51.6)	=0.004
Age, mean (SD)	34.7 (6.2)	34.5 (6.6)	33.3 (5.8)	34.5 (6.2)	36.0 (5.6)	35.4 (6.3)	=0.009
Educational level, N (%)							<0.001
Tertiary	163 (7.5)	16 (3.6)	22 (4.5)	25 (6.8)	29 (6.6)	71 (15.8)	
Secondary	1106 (50.7)	190 (42.9)	398 (82.1)	84 (22.8)	117 (26.7)	318 (71.0)	
Lower than secondary	911 (41.8)	237 (53.5)	65 (13.4)	259 (70.4)	292 (66.7)	59 (13.2)	
Wealth tertiles, N (%)							<0.001
High	557 (26.5)	146 (33.0)	162 (32.9)	91 (24.7)	112 (25.6)	67 (15.0)	
Middle	691 (31.7)	141 (31.8)	150 (33.4)	101 (27.5)	161 (36.8)	139 (31.0)	
Low	912 (41.8)	156 (35.2)	173 (31.0)	176 (47.8)	165 (37.7)	242 (54.0)	
Smoking, N (%)	571 (26.2)	11 (2.5)	167 (34.4)	92 (25.0)	91 (20.8)	210 (46.9)	<0.001
Physically active, N (%)	853 (39.1)	219 (49.4)	233 (48.1)	107 (29.1)	179 (40.9)	115 (25.7)	<0.001
Obese, N (%)	665 (30.5)	46 (10.4)	156 (32.2)	115 (31.3)	110 (25.1)	238 (53.1)	<0.001
Hypertensive, N (%)	290 (13.3)	20 (4.5)	96 (19.8)	25 (6.8)	53 (12.1)	96 (21.4)	<0.001
High cholesterol, N (%)	336 (15.4)	55 (12.4)	52 (10.7)	46 (12.5)	71 (16.2)	112 (25.0)	<0.001
Elevated blood glucose, N (%)	686 (31.5)	NA	44 (9.1)	74 (20.1)	168 (38.4)	184 (41.1)	<0.001

^a^ *p* value for difference across sites

^b^Gross National Income per capita in US dollars ($)

^c^Human Development Index (2010)

^d^HDI and world income class are reported for the country from which the five populations are drawn, but these indexes may be different for the specific communities sampled in METS

**Fig. 1:**
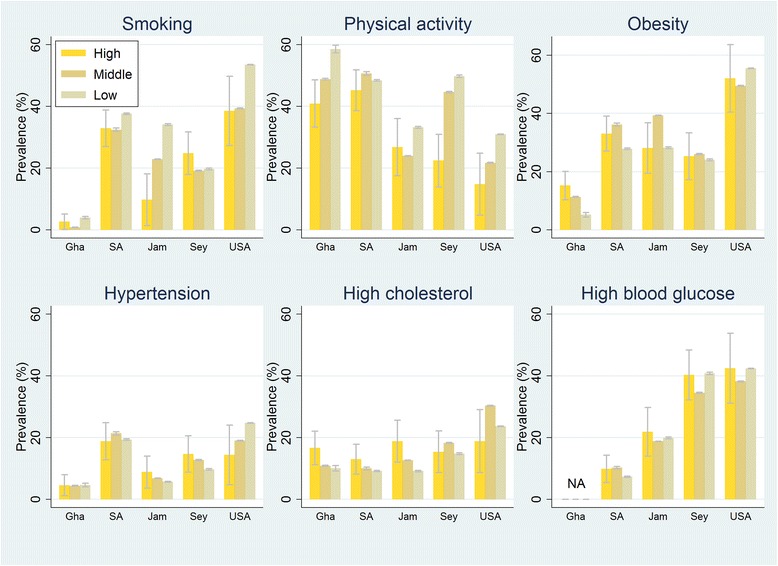
Age and sex adjusted prevalence of risk factors of noncommunicable disease in five sites, by education level

Age-and sex-adjusted prevalence rates of NCD-RFs in the five sites according to *educational level* are presented in Fig. 1. Smoking was more prevalent among participants with a low education in three sites, but was not patterned by education in the South African and Ghanaian samples. The prevalence of physical activity was higher among individuals with low education in most sites apart from South Africa. Obesity was more prevalent among individuals with middle education in all sites; hypertension among individuals with low education in the USA, Ghanaian and Jamaican samples, and among those with high education in the South African and Seychelles samples. High cholesterol was not strongly patterned by education except for the Jamaican sample (more prevalent among highly educated individuals). The prevalence of elevated blood glucose was higher among lower educated individuals in the USA and Seychelles’ samples.

**Fig. 2:**
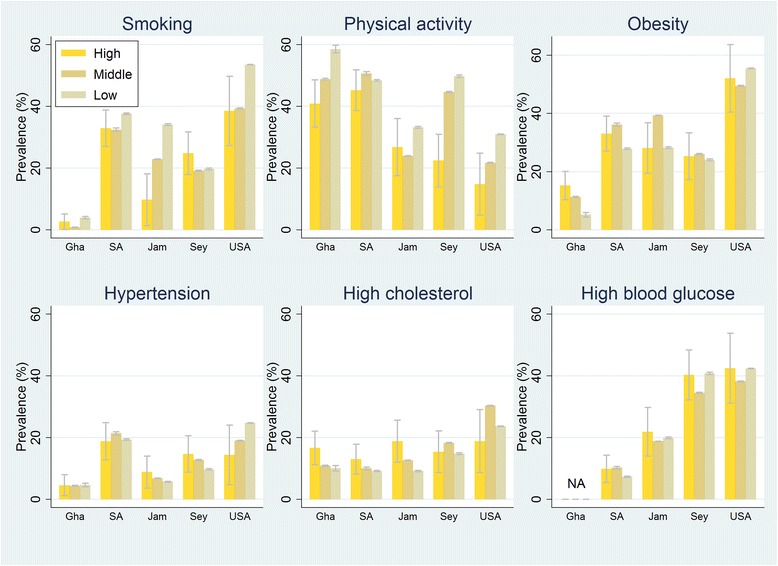
Age and sex adjusted prevalence of risk factors of noncommunicable diseases in five sites, by wealth

Figure 2 shows age-and sex-adjusted prevalence rates by *wealth tertiles*. Results were generally similar to those observed for education for current smoking and physical activity. Obesity was more prevalent among individuals in the middle wealth tertile in the South African and Jamaican populations and was not patterned by wealth in the USA sample. In the Ghanaian sample, an inverse gradient was observed (higher obesity rates with increasing wealth). The prevalence of hypertension was higher among individuals in the lowest wealth tertile in the USA and among those in the highest tertile in the Seychelles. High cholesterol was more prevalent among the wealthier individuals in the South African, Ghanaian and Jamaican samples. Elevated blood glucose did not seem to be patterned by wealth.

Table 2 shows results for the association of education and wealth with NCD-RFs by site. Smoking was more likely among individuals with low SES in the USA and Jamaican’s samples (*p* < 0.01) and was not patterned by SES in Ghanaian sample. Physical activity was more likely among individuals with low education and wealth in all sites and for both genders (*p* < 0.05 for all sites but South Africa). Obesity was less likely among low SES individuals in the Ghanaian sample (*p* = 0.004 for wealth). In the USA sample, individuals with high education tended to be more frequently obese, but wealthier women were less likely to be obese. Low education tended to be associated with higher obesity but no clear pattern was observed for wealth in the Seychelles’ sample, while middle SES individuals were generally more likely to be obese in the South African and Jamaican samples. Hypertension was more likely among high SES individuals in the USA (*p* = 0.048 for wealth), and was not clearly patterned by SES in South African sample. In Ghana and Jamaica population samples, hypertension tended to be more likely among individuals with low SES, and in the Seychelles among individuals with high SES. High cholesterol was more likely among high SES individuals in the Jamaican sample (*p* = 0.026), and tended to be more likely in the middle SES group in the USA and the Seychelles’ samples. In the South African sample, high education tended to be associated with lower cholesterol levels but higher wealth with higher levels. Elevated blood glucose was more likely among the less educated individuals in the USA (*p* = 0.007) and Seychelles’s samples (*p* > 0.10). Elevated blood glucose tended to be more likely among higher educated individuals in the Jamaican sample, and there was no clear association in South Africa.

**Table 2:** Association of education and wealth with risk factors of noncommunicable diseases by site, age 25–45 years, Ghana (*N* = 438), South Africa (*N* = 484), Jamaica (*N* = 323), Seychelles (*N* = 438), United States (*N* = 448)

	Education					Wealth				
	High	Middle	Low	*p* ^*trend*a^	*DIR* ^*b*^	High	Middle	Low	*p* ^*trend*a^	*DIR* ^*b*^
Smoking										
Ghana	1.00	0.34 (0.03–3.61)	0.69 (0.07–6.5)	0.657	+	1.00	0.26 (0.03–2.34)	1.74 (0.47–6.43)	0.481	-
South Africa	1.00	0.70 (0.23–2.12)	0.81 (0.23–2.82)	0.983	+	1.00	0.95 (0.52–1.72)	1.37 (0.79–2.40)	0.261	-
Jamaica	1.00	2.29 (0.47–11.21)	4.62 (1.04–20.6)	0.005	-	1.00	2.73 (1.18–6.3)	5.12 (2.39–10.96)	<0.001	-
Seychelles	1.00	1.30 (0.42–4.03)	1.30 (0.44–3.87)	0.740	-	1.00	0.67 (0.35–1.3)	0.68 (0.36–1.30)	0.281	+
USA	1.00	5.28 (2.39–11.67)	47.5 (16.2–139.2)	0.000	-	1.00	1.04 (0.55–1.95)	1.97 (1.10–3.53)	0.003	-
Physical activity										
Ghana	1.00	1.81 (0.61–5.44)	2.02 (0.68–6.00)	0.300	-	1.00	1.48 (0.89–2.47)	2.37 (1.41–3.99)	0.001	-
South Africa	1.00	1.75 (0.63–4.88)	1.27 (0.40–4.02)	0.853	-	1.00	1.36 (0.8–2.32)	1.20 (0.72–1.99)	0.505	-
Jamaica	1.00	1.04 (0.34–3.21)	1.87 (0.67–5.21)	0.045	-	1.00	0.86 (0.44–1.65)	1.37 (0.78–2.42)	0.187	-
Seychelles	1.00	1.22 (0.50–2.97)	1.67 (0.72–3.88)	0.109	-	1.00	3.07 (1.74–5.42)	3.84 (2.18–6.76)	<0.001	-
USA	1.00	1.23 (0.60–2.55)	3.11 (1.32–7.32)	0.005	-	1.00	1.63 (0.73–3.65)	2.74 (1.30–5.76)	0.002	-
Obesity										
Ghana	1.00	1.21 (0.14–10.41)	1.03 (0.12–8.87)	0.726	NA	1.00	0.62 (0.29–1.31)	0.29 (0.13–0.68)	0.004	+
South Africa	1.00	1.84 (0.57–5.91)	1.11 (0.28–4.41)	0.720	-	1.00	1.19 (0.67–2.12)	0.71 (0.40–1.27)	0.223	+
Jamaica	1.00	1.89 (0.64–5.56)	1.75 (0.63–4.82)	0.518	-	1.00	1.88 (0.95–3.72)	1.02 (0.55–1.91)	0.717	-
Seychelles	1.00	1.60 (0.59–4.31)	1.27 (0.49–3.28)	0.847	-	1.00	1.05 (0.6–1.83)	0.93 (0.53–1.64)	0.781	NA
USA	1.00	1.05 (0.60–1.82)	0.61 (0.30–1.27)	0.207	+	1.00	0.90 (0.49–1.64)	1.16 (0.66–2.03)	0.391	-
Hypertension										
Ghana	NA	1.00	1.68 (0.65–4.36)	0.286	-	1.00	0.95 (0.29–3.05)	1.00 (0.32–3.10)	0.988	NA
South Africa	1.00	0.60 (0.22–1.65)	0.85 (0.28–2.62)	0.673	+	1.00	1.18 (0.66–2.12)	1.03 (0.59–1.81)	0.918	NA
Jamaica	1.00	1.82 (0.21–15.88)	1.80 (0.23–14.14)	0.688	-	1.00	0.76 (0.26–2.18)	0.62 (0.24–1.63)	0.338	+
Seychelles	1.00	0.94 (0.30–2.91)	0.80 (0.27–2.38)	0.577	+	1.00	0.84 (0.41–1.74)	0.62 (0.29–1.32)	0.214	+
USA	1.00	1.75 (0.81–3.79)	1.89 (0.74–4.86)	0.199	-	1.00	1.39 (0.62–3.12)	1.96 (0.93–4.12)	0.048	-
High cholesterol										
Ghana	1.00	0.86 (0.18–4.13)	0.83 (0.17–3.92)	0.835	+	1.00	0.57 (0.28–1.17)	0.54 (0.27–1.10)	0.090	+
South Africa	1.00	2.64 (0.35–20.09)	2.41 (0.28–21.04)	0.685	-	1.00	0.75 (0.37–1.52)	0.68 (0.34–1.35)	0.267	+
Jamaica	1.00	0.41 (0.13–1.29)	0.41 (0.15–1.11)	0.178	+	1.00	0.62 (0.28–1.38)	0.43 (0.20–0.90)	0.026	+
Seychelles	1.00	1.59 (0.49–5.15)	1.50 (0.48–4.66)	0.704	-	1.00	1.25 (0.65–2.40)	0.96 (0.49-1.88)	0.829	NA
USA	1.00	1.89 (0.94–3.77)	0.84 (0.32–2.20)	0.832	NA	1.00	1.92 (0.93–3.93)	1.33 (0.67–2.65)	0.912	-
Elevated blood glucose										
Ghana	NA					NA				
South Africa	1.00	1.00 (0.22–4.5)	1.00 (0.19–5.34)	0.999	NA	1.00	1.04 (0.49–2.21)	0.72 (0.33–1.56)	0.408	+
Jamaica	1.00	0.58 (0.19–1.85)	0.68 (0.24–1.90)	0.756	+	1.00	0.78 (0.38–1.64)	0.87 (0.46–1.66)	0.726	+
Seychelles	1.00	1.78 (0.68–4.69)	1.58 (0.63–4.00)	0.692	-	1.00	0.73 (0.41–1.27)	1.03 (0.59–1.78)	0.785	NA
USA	1.00	1.80 (0.97–3.35)	2.92 (1.34–6.35)	0.007	-	1.00	0.82 (0.44–1.52)	0.99 (0.56–1.74)	0.797	NA

*NA* indicates no association. We also consider here also trends towards association, i.e., marginally significant *p* values (*p* > 0.05& *p* < 0.09)

^a^p for linear trend across socio-economic categories

^b^Direction of social differences

+ indicates that the prevalence of the NCD-RF is higher among higher SES groups, − indicates that the prevalence is higher among lower SES groups

The association between SES indicators and NCD-RFs significantly differed by site for smoking, physical activity and elevated blood glucose (*p* < 0.05). Education did not seem to modify the association between wealth and NCD-RFs, with the exception of obesity in the USA sample, where the association between wealth and obesity was stronger among individuals with a higher educational level (p for interaction = 0.030).

## Discussion and Conclusions

In this study, we examined the social patterning of several NCD-RFs in five populations from countries at different levels of the epidemiological transition. The distribution of NCD-RFs was substantially patterned by education and wealth, with individuals with less education or wealth generally showing a higher prevalence of several NCD-RFs. However, associations differed substantially according to NCD-RFs, population, sex, and SES indicator. Smoking and high physical activity were associated with low SES in most countries. Obesity, hypertension, cholesterol and elevated blood glucose had a different social distribution by country, sex, and SES indicator. For example, the prevalence of elevated blood glucose tended to be associated with low education, but not with low wealth, in countries at an advanced stage of the epidemiologic transition (the Seychelles and the USA). The association of SES with obesity and cholesterol tended to be direct in some countries but inverse in others. The prevalence of some NCD-RFs seemed to be higher in intermediate SES categories, especially in populations from countries at an intermediate stage of the epidemiologic transition (e.g., obesity in South Africa, Jamaica and Seychelles).

Inconsistencies in the social patterning of NCD-RFs by risk factor, population, sex, and SES indicator have been observed in virtually all multi-country studies in LMICs [3–6, 18]. Our results, based on data from five populations recruited using standardized data collection methods, support the notion that the association between SES indicators and NCD-RFs changes along the epidemiological transition, at different paces for men and women (generally more rapidly in men) and differently for different risk factors (e.g., earlier for smoking). A major implication of these results is the need to assess and integrate the social patterning of NCD-RFs in NCD surveillance and in prevention and control programs in LMICs. In particular, we emphasize the need to routinely collect data according to several socio-economic categories and to monitor the progression of the social distribution of NCDs and their risk factors over time.

A strong social patterning of smoking was observed in most sites, as expected. Remarkably, nearly all less educated men were current smokers in the USA, likely reflecting a more disadvantaged community than the general American population. In the South African sample, higher wealth seemed to be protective against smoking, while highly educated women were more likely to smoke. These results partly contrast with previous studies reporting an inverse association of income and education with current smoking in the adult South African population [19], and higher smoking cessation rates among highly educated women [20]. The positive association between education and smoking that we observed among women may be explained by the relatively young age of our study population. The low prevalence of smoking in Ghana (with virtually no smoking women) and its association with education among men suggests that rural Ghana may still be at a very early stage of the smoking epidemics [21].

### Physical activity and obesity

Social differences in physical activity were examined in a previous paper on this population [22]. We found comparable levels of physical activity across sites, and an inverse association between SES and physical activity for both genders, with the exception of the South African sample where the middle SES groups had the highest prevalence of physical activity. The fact that high SES individuals were more likely to own a car in the five sites (and thus presumably move less) is a plausible explanation for higher physical activity levels in the lower SES groups [22]. Moreover, low SES individuals are more likely to be employed in activity-intense jobs. Our results are consistent with previous international reports on the association between SES and physical activity in LMICs [23, 24]. However, in the USA a positive association between SES and physical activity is generally observed [25, 26], as in most high income countries, while in our study physical activity levels were higher among low SES individuals. These differences are probably related to the fact that low SES individuals in this disadvantaged population subgroup were less likely to own a car and had a longer commuting to work compared to individuals of low SES in the general population of the country [27].

The prevalence of obesity was higher in women than men, as commonly found in African American populations [28, 29]. Studies assessing social differences in obesity among African American women have yielded conflicting results [30, 31]. In our study, lower education was associated with higher prevalence of obesity in countries at an intermediate stage of the epidemiologic transition (South Africa and the Seychelles), but the reverse was observed in the Ghanaian and the USA’s sample. Rural Ghana, from which our sample was drawn, is probably at an early stage of the epidemiologic transition. Results for the USA sample are consistent with findings for physical activity and maybe explained by the peculiarities of this particular population, sampled in the Chicago suburbs, as a negative association between education and obesity is generally found in the USA [32]. Previous studies have suggested that education and material resources interact in shaping the distribution of obesity in LMIC settings [6, 11]. In our study, education did not modify the association between wealth and obesity in African countries, although we had limited power to detect significant interactions. In this US population from a Chicago neighbourhood, high education seemed to confer an even greater obesity risk to individuals in the lowest wealth tertile. Inconsistencies with results from other studies may be related to the possibility that the American population from a Chicago suburb that participated in this study is not representative of all African-Americans. In rural Ghana, women with high SES had a higher prevalence of obesity (particularly those in the highest wealth tertile), in contrast with a previous study that reported a positive association between SES and obesity in men and an inverse association in women [33]. These divergences suggest that social influences on NCD-RFs may differ in urban and rural areas in LMICs. In South Africa and Jamaica, obesity rates were higher in the middle SES groups, consistent with intermediary stages of secular shifts in the social patterning of obesity.

### Hypertension, high cholesterol and elevated blood glucose

Hypertension was strongly socially patterned in the USA, participants with low SES having a significantly higher prevalence relative to those with high SES. This pattern has been frequently observed in high income countries [34–36]. Differences in treatment rates or access to health care may partly explain this association, along with trends towards higher salt intake, lower potassium intake, greater BMI, and higher alcohol intake in low SES individuals [36, 37]. Social differences in hypertension were less pronounced in other sites in our study, as expected given that a mix of positive and negative gradients has been found across studies in sub-Saharan Africa [38–42].

In countries at a later stage of the epidemiologic transition (USA and Seychelles), high blood cholesterol and glucose were generally more prevalent among low SES groups, as reported in other studies [35, 43]. Poorer nutrition and higher BMI are potential mediators of these associations; although in the USA the prevalence of obesity tended to be higher among higher SES groups. In countries at intermediate/early stages of the epidemiologic transition, individuals with a low wealth tended to have lower prevalence of high cholesterol and elevated glucose, consistent with higher lipid levels generally found among people from middle or higher SES groups in many transitional countries [44]. Larger consumption of a diet rich in saturated fat and reduced physical activity among the wealthier persons may explain these associations in LMICs.

### Strengths and limitations

This is one of the few studies to examine the association of two indicators of SES with several NCD-RFs in LMICs, using validated questionnaires and standardized measurements in samples of several populations at different levels of the epidemiological transition. The relatively young age of the participants also allows assessing the social determinants of NCD-RFs before NCDs have developed, which limits interference of treatments (and their social distribution). This study also has several limitations. First, the sample per site was fairly small, limiting the analysis of gender-specific associations and interactions. Subsequently, several associations showed large effect sizes but non-significant *p* values, likely related to insufficient statistical power. However, given the scarcity of data on the SES- NCD-RF associations in LMICs, this study is useful for informing public health strategies. Second, the study populations represent specific communities in most study sites, which are not representative of their respective national populations. The specificities of some of these communities may explain some inconsistencies between our results and those of previous studies, especially in the USA. Third, our wealth variable was constructed using a simple method assigning equal weights to all owned assets within and between countries to account, while some assets may have more values than others. Finally, although our cross sectional data on the social distribution of NCD-RFs in countries at different level of socioeconomic development may inform on future trends, only repeated surveys allow monitoring the actual social transition of risk factors over time in each population. Further sufficiently powered studies, allowing analyses by sex, age and SES status, should assess trends in the social patterning of NCD-RFs in a range of countries of vastly different socio-economic development, using repeated independent cross-sectional surveys.
